# Multi-User Opportunistic Spectrum Access for Cognitive Radio Networks Based on Multi-Head Self-Attention and Multi-Agent Deep Reinforcement Learning

**DOI:** 10.3390/s25072025

**Published:** 2025-03-24

**Authors:** Weiwei Bai, Guoqiang Zheng, Weibing Xia, Yu Mu, Yujun Xue

**Affiliations:** 1 College of Information Engineering, Henan University of Science and Technology, Luoyang 471023, China; baiww@stu.haust.edu.cn (W.B.); muyuvlc2018@foxmail.com (Y.M.); 2 Luoyang Artificial Intelligence Research Institute Co., Ltd., Luoyang 471000, China; xwb@lyict.ac.cn; 3 School of Mechatronics Engineering, Henan University of Science and Technology, Luoyang 471023, China; yjxue@haust.edu.cn

**Keywords:** opportunistic spectrum access, throughput, multi-head self-attention, multi-agent deep reinforcement learning, multi-user spectrum access, cognitive radio network

## Abstract

Aiming to address the issue of multi-user dynamic spectrum access in an opportunistic mode in cognitive radio networks leading to low sum throughput, we propose a multi-user opportunistic spectrum access method based on multi-head self-attention and multi-agent deep reinforcement learning. First, an optimization model for joint channel selection and power control in multi-user systems is constructed based on centralized training with a decentralized execution framework. In the training phase, the decision-making policy is optimized using global information, while in the execution phase, each agent makes decisions according to its observations. Meanwhile, a multi-constraint dynamic proportional reward function is designed to guide the agent in selecting more rational actions by refining the constraints and dynamically adjusting the reward proportion. Furthermore, a multi-head self-attention mechanism is incorporated into the critic network to dynamically allocate attention weights to different users, thereby enhancing the ability of the network to estimate the joint action value. Finally, the proposed method is evaluated in terms of convergence, throughput, and dynamic performance. Simulation results demonstrate that the proposed method significantly improves the sum throughput of secondary users in opportunistic spectrum access.

## 1. Introduction

With the rapid increase in wireless devices and the gradual advancement of 6G networks, the demand for wireless services has surged explosively. This demand further exacerbates the scarcity of wireless spectrum resources. Under the traditional static spectrum allocation policy, licensed users possess exclusive rights to specific frequency bands. However, the utilization rate of these spectrum resources tends to be low, resulting in significant instances of idle or underutilized spectra. This inefficient spectrum usage stands in stark contrast to the dramatic increase in demand for wireless services, leading to a serious waste of spectrum resources. To address this challenge, cognitive radio technology has emerged as a viable solution. As a crucial component of 6G networks, cognitive radio networks are capable of dynamically identifying and utilizing spectrum resources [1,2]. Based on this, the opportunistic spectrum access (OSA) model is proposed, which enables other users to dynamically access and reuse spectrum resources when licensed users are not utilizing them, thereby enhancing spectrum utilization and mitigating waste [3,4,5,6]. In the OSA model, the user with exclusive rights to a particular frequency band is referred to as the primary user (PU), while the user that opportunistically accesses the spectrum is known as the secondary user (SU). The SU aims to access unoccupied licensed frequency bands to enable spectrum sharing. To prevent interference with PUs, SUs must periodically perform spectrum sensing to detect channel availability. The design of opportunistic spectrum access policies presents several challenges, including dynamic and unpredictable communication environments, interactions among multiple SUs, and the impact of communication requirements on access decisions [7].

To improve spectrum utilization, many researchers have investigated the problem of opportunistic spectrum access [8,9]. The spectrum sensing issues related to opportunistic spectrum access were explored in [10,11,12,13,14]. Specifically, spectrum detection methods based on Support Vector Machines (SVMs) were proposed in [10,11]. The K-means clustering method was employed in [12] for spectrum sensing. Convolutional Neural Networks (CNNs) were utilized in [13] to extract features from spectrum data, enhancing sensing accuracy. Additionally, the study in [14] combined CNNs with long short-term memory (LSTM) networks to further improve sensing performance. However, spectrum access involved not only spectrum sensing but also the dynamic allocation and coordination of spectrum resources among multiple SUs, making it an inherently complex optimization problem that traditional neural networks were poorly equipped to address. Cooperative spectrum sharing between multiple PUs and SUs was modeled as a two-sided matching market in [15], addressing the pairing problem between PUs and SUs to optimize the allocation of PU access time. A polynomial time-based many-to-one matching scheme was proposed to foster cooperation among SUs and enhance overall efficiency. However, the scheme relied on the assumption that collaborative communication was feasible between PUs and SUs. A queuing policy incorporating a retry mechanism was proposed in [16], which aimed to improve spectrum access efficiency in cognitive radio networks (CRNs), thereby increasing system throughput and spectrum utilization. In the network scenario discussed in [16], a central controller collected information from both PUs and SUs and made dynamic channel access decisions accordingly. The power control and spectrum access problem of SUs in uplink opportunistic CRNs was investigated in [17], where a parameterized power control policy was proposed that allowed SUs to adaptively adjust their transmit power based on channel fading information and battery energy status feedback from the access point (AP), thereby maximizing the total uplink rate between SUs and AP. However, the study did not consider the mobility of the SUs. These methods usually rely on mathematical models and require predefined rules and parameters. However, spectrum access and channel conditions in future network environments will be more complex and dynamic, demanding more flexible and efficient solutions.

In recent years, deep reinforcement learning (DRL) algorithms have been introduced into OSA due to their capacity to adapt to unknown environments [18,19]. The study in [20] addressed the issue of opportunistic access in multi-channel heterogeneous networks (HetNets) and proposed a novel multi-channel MAC protocol to optimize wireless channel access policies, thereby improving network throughput. The study employed a DRL technique, which combined a deep Q-network (DQN) with a gated recurrent unit (GRU). This combination allowed the GRU to effectively capture temporal features in multi-channel HetNets and supported efficient decision-making in dynamic environments. In [21], a spectrum handoff algorithm based on DQN was designed. Additionally, migration learning was introduced to accelerate the learning process for newly joined SUs, reducing both the time and computational costs associated with initial exploration. The algorithm took into account the priority of SUs and assumed that each channel could only be assigned to one user at a time. Two deep recurrent Q-network (DRQN) architectures based on long short-term memory were proposed to address user conflicts and throughput loss in opportunistic spectrum access in [22]. The collisions between users were minimized by exploiting the temporal correlation of SU transmissions. Simulation results demonstrated that the proposed DRQN performed well, offering a complexity–performance trade-off for scenarios with partial observations and multiple channels. However, the study did not take into account the changing channel and relative location of the users, whereas, in practice, these communication environments are dynamically changing. The work mode selection and continuous power allocation problem for cognitive nodes was addressed in [23], where a method based on the deep deterministic policy gradient (DDPG) was proposed, and its effectiveness was verified through simulations. However, the method did not consider scenarios in which multiple nodes could access a channel, as it assumed that each channel allowed only one node to access it.

Furthermore, the interaction among multiple SUs is also a challenge. The objective of SUs is to opportunistically access an idle channel for data transmission. However, in a multi-user network, the spectrum access choices of SUs may lead to channel contention and interference, which can cause SUs to interact with each other in their behavior. Independently optimizing the policy of each SU may lead to system instability, and the observation information of a single SU is insufficient to fully reflect the current network state. On the other hand, the centralized training and execution approach increases the dimensionality of decision-making and reliance on global information, which is detrimental to the scalability and real-time performance of the network.

In summary, the system models in existing opportunistic spectrum access methods typically do not take into account changes in the relative positions of users, assume that each channel can be used by only one SU, or simplify the interaction between SUs. These premises may make it difficult for methods to cope with the various challenges in dynamic environments. To address these limitations, this paper proposes a novel OSA model. Specifically, the proposed model incorporates the relative positional changes of users, allows multiple SUs to share the same channel, and considers more complex interactions among SUs. Based on this, a multi-user opportunistic spectrum access method based on multi-head self-attention and multi-agent deep reinforcement learning (MSA-MADRL-OSA) is proposed. This method adopts a centralized training with decentralized execution framework and incorporates a multi-head self-attention (MSA) mechanism to optimize the spectrum access policy. The main contributions of this paper are as follows:

- A new OSA model is established. In this model, each SU moves randomly within the designated area in each time slot and selects a channel for access based on spectrum sensing results. Since multiple SUs are allowed to utilize an idle channel, channel contention and interference may occur among SUs sharing the same channel. While ensuring that collisions with PUs are avoided, SUs strive to transmit as much data as possible to maximize the sum throughput of the SUs.
- A joint channel selection and power control optimization framework based on multi-agent deep reinforcement learning (MADRL) is proposed. Each SU is treated as an agent, whose actions are joint channel selection and power control. A centralized training with decentralized execution framework is adopted, where global information is used to optimize the decision-making policy during the training phase. In the execution phase, each SU only relies on its observation information to make decisions, which improves the scalability and real-time performance of the system. Meanwhile, a multi-constraint dynamic proportional reward function is designed, which refines the constraints and dynamically adjusts the reward proportion to guide the agent in selecting more rational actions.
- An MSA-MADRL-OSA method is proposed. In this method, an MSA mechanism is incorporated into the critic network, enhancing the ability of the critic network to estimate the action value by dynamically allocating attention weights to different SUs. This enables the SUs to efficiently learn and adapt to dynamic changes in the complex multi-agent environment and optimize the spectrum access policy.
- The performance of the proposed method is evaluated in terms of convergence, throughput, and dynamic performance. The simulation results indicate that the proposed method enables SUs to make rational spectrum access decisions, thereby improving the sum throughput of SUs.

## 2. System Model

This section presents the construction and analysis of the system model and the channel transmission performance model, focusing on the basic structure of the system and the impact of channel characteristics on the transmission performance.

### 2.1. System Model Description

**Figure 1:**
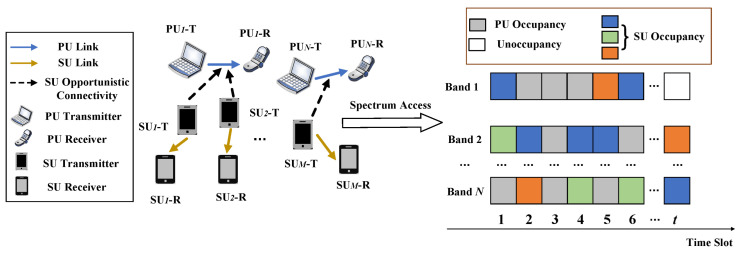
Model of multi-user opportunistic spectrum access.

We consider a cognitive radio network dynamic spectrum access scenario consisting of *N* primary users and *M* secondary users, with PUs and SUs denoted by $PU_{n}\left(n=1,2,\cdots ,N\right)$ and $SU_{m}\left(m=1,2,\cdots ,M\right)$, respectively. In Figure 1, each user consists of a transmitter and a receiver. The PUs and SUs operate in a clock-synchronized manner, the channel state of the PUs and the channel access of the SUs are updated once for each time slot, and no cooperative communication takes place between the PUs and SUs. Each PU is assigned a licensed channel, and all channels are mutually orthogonal, meaning that each PU transmits on its channel without interference between channels. In addition, the busy and idle periods of each channel are respectively given by the independent random processes, and the arrival rate of the $PU_{n}$ on channel *n* is a Poisson process [9]. The SUs share the channels of the PUs in opportunistic spectrum access mode. In this mode, the PUs have priority use of the channels, and the SUs can access them for data transmission only when the channels of the PUs are idle. The SU senses the spectrum state of the PU to select the idle channel to access. Let the spectrum state sensed by the $SU_{m}$ at time slot *t* be represented as $Z_{m}\left(t\right)=\left[z_{m1}\left(t\right),z_{m2}\left(t\right),\cdots ,z_{mN}\left(t\right)\right]$, where $z_{mn}\left(t\right)\in \left\{0,\left.1\right\}\right.,n=1,2,\cdots ,N$, and $z_{mn}\left(t\right)=1$ indicates that channel *n* is occupied by the $PU_{n}$, while $z_{mn}\left(t\right)=0$ indicates that it is unoccupied.

### 2.2. Channel and Transmission Performance Model

In the system, the large-scale path loss between different transmitters and a receiver is calculated by the WINNER II channel model [24]:

(1) ξdB=C0+βlog10da,b+Bξlog10fcfc55+ξ0,

where $C_{0}$ is a constant term for the path loss whose value is related to the environmental conditions, β is the path loss exponent, $d_{a,b}$ is the distance between the transmitter *a* and the receiver *b*, $B_{\xi }$ is the frequency attenuation coefficient, $f_{c}$ is the carrier frequency, and $\xi_{0}$ denotes the path loss at the reference distance. The variance of the channel gain denoted as σ2=10−ξξ1010. Then, the variation of channel gain $h_{a,b}$ from transmitter *a* to receiver *b* is modeled by the Rician distribution:

(2) $$h_{a,b}=\sqrt{\frac{k}{k+1}}\sigma e^{j\phi }+\sqrt{\frac{1}{k+1}}CN\left(0,\sigma^{2}\right),$$

where $CN\left(0,\sigma^{2}\right)$ is a complex Gaussian random variable with mean 0 and variance $\sigma^{2}$, *k* is the Rician factor, and $\phi ∼u\left(0,2\pi \right)$ is the uniformly distributed random phase of the arrival signal. Furthermore, in this paper, it is assumed that the channel gain remains constant within a time slot but changes dynamically between time slots.

Additionally, this paper assumes that each SU can use only one channel for data transmission in each time slot, but a channel may be used simultaneously by multiple SUs. We denote the transmit power of the $SU_{m}$ by $p_{m}^{S}\left(t\right)$, the noise power spectral density by $N_{0}$, and the bandwidth of each channel by *W*. If the PU on channel *n* is represented as $PU_{n}$, three possible situations can occur when the $SU_{m}$ selects channel *n* for transmission in time slot *t*. These situations will affect the signal-to-noise ratio (SNR) calculation at the $SU_{m}$ receiver.

- No $PU_{n}$ is present on the channel, and only the $SU_{m}$ transmits on channel *n*. In this situation, the SNR at the $SU_{m}$ receiver is calculated as [25]
  (3) $$\gamma_{m}^{S}\left(t\right)=\frac{{\left|h_{S_{m}T,S_{m}R}\left(t\right)\right|}^{2}p_{m}^{S}\left(t\right)}{WN_{0}},$$
  where $h_{S_{m}T,S_{m}R}\left(t\right)$ represents the channel gain from the transmitter to the receiver of $SU_{m}$.
- No $PU_{n}$ is present on the channel, but multiple SUs have selected channel *n* for transmission. In this situation, the SNR at the $SU_{m}$ receiver is calculated as [26]
  (4) $$\gamma_{m}^{S}\left(t\right)=\frac{{\left|h_{S_{m}T,S_{m}R}\left(t\right)\right|}^{2}p_{m}^{S}\left(t\right)}{WN_{0}+\sum_{e\neq m}{\left|h_{S_{e}T,S_{m}R}\left(t\right)\right|}^{2}p_{e}^{S}\left(t\right)},$$
  where $\sum_{e\neq m}{\left|h_{S_{e}T,S_{m}R}\left(t\right)\right|}^{2}p_{e}^{S}\left(t\right)$ is the sum of the products of the channel gains from the transmitters of other SUs on channel *n* to the receiver of the $SU_{m}$ and the transmit powers of those SUs.
- The $PU_{n}$ transmits on channel *n*, resulting in an interruption of the $SU_{m}$ transmission. Therefore, the SNR at the $SU_{m}$ receiver cannot be calculated.

In addition, the transmission of $SU_{m}$ must satisfy the minimum SNR $\gamma_{th}$ for successful transmission. The throughput of $SU_{m}$ is calculated as [27]

(5) $$R_{m}^{S}\left(t\right)=W\log_{2}\left(1+\gamma_{m}^{S}\left(t\right)\right).$$

## 3. Problem Statement

Based on the aforementioned system model, to reasonably utilize limited channels for transmission and enhance overall throughput, SUs must adhere to the relevant constraints during dynamic channel access to ensure that PUs maintain priority in spectrum usage. Therefore, the multi-user opportunistic spectrum access problem can be described as follows: given uncertainties in the initial state, system model, and environmental parameters, the goal is to maximize the sum throughput of the SUs by designing effective channel selection and power control policies for SUs, while satisfying constraints such as PU channel priority and minimum SNR for SUs.

In multi-user opportunistic spectrum access, each SU needs to select both a channel and a transmit power within each time slot, combining these actions into a joint action. If the channel access of the $SU_{m}$ is denoted as $\left(c_{m}\left(t\right),p_{m}^{S}\left(t\right)\right)$, where $c_{m}\left(t\right)$ is the channel choice of the $SU_{m}$ and $p_{m}^{S}\left(t\right)$ is the transmit power of the $SU_{m}$, then

(6) $$c_{m}\left(t\right)\in \left\{0,1,\cdots ,N\right\},$$

(7) $$p_{m}^{S}\left(t\right)\in \left[p_{\min},p_{\max}\right],$$

where $c_{m}\left(t\right)=0$ represents that the SU does not select the channel to transmit data, and $p_{\min}$ and $p_{\max}$ denote the minimum and maximum transmit power of the $SU_{m}$, respectively.

To maximize the sum throughput of SUs, this paper formulates the multi-user opportunistic spectrum access problem as an optimization problem with multiple constraints, expressed as

(8) $$\begin{array}{l} P:\max\sum_{t=1}^{T}\sum_{m=1}^{M}R_{m}^{S}\left(t\right)\\ s.t.\\ \;C1:c_{m}\left(t\right)\in \left\{0,1,\cdots ,N\right\},m=1,2,\cdots ,M\\ \;C2:p_{\min}⩽p_{m}^{S}\left(t\right)⩽p_{\max},m=1,2,\cdots ,M\\ \;C3:\gamma_{m}^{S}\left(t\right)⩾\gamma_{th},m=1,2,\cdots ,M\\ \;C4:z_{mn}\left(t\right)\in \left\{0,1\right\},m=1,2,\cdots ,M,n=1,2,\cdots ,N. \end{array}$$

According to Equation  (8), the optimization objective is to maximize the sum throughput of all SUs over *T* time slots. C1 constrains each SU to select at most one channel transmission each time slot. C2 constrains the transmit power of the SU. C3 constrains the minimum SNR, $\gamma_{th}$, for a successful transmission by an SU. C4 constrains the channel to an unoccupied or occupied state.

Multiple SUs simultaneously access limited channel resources, and the interaction between their transmit power and channel selection adds complexity to the problem. Solving this optimization problem requires a priori information, such as the spectrum access mechanism of the SUs and accurate channel states, which are difficult to obtain in practice, leading to the fact that the traditional solution methods are no longer applicable. Therefore, in this paper, a multi-agent deep reinforcement learning (MADRL) method is used to solve this problem.

## 4. Multi-Agent Deep Reinforcement Learning-Based Multi-User Opportunistic Spectrum Access (MADRL-OSA) Method

In the OSA scenario, the objective of the SUs is to effectively utilize the unoccupied spectrum resources of the PUs for data transmission through dynamic spectrum sensing and decision-making. However, in a multi-user competitive environment, the decision-making behaviors of multiple SUs will affect each other, which may lead to system performance degradation or even instability if independent optimization strategies are used. Furthermore, in the framework of a partially observable Markov decision process, a single agent can only obtain local observation information, which complicates the accurate assessment of the overall state of the system. To address the aforementioned challenges, we propose an opportunistic spectrum access method for MADRL based on a centralized training with decentralized execution framework.

**Figure 2:**
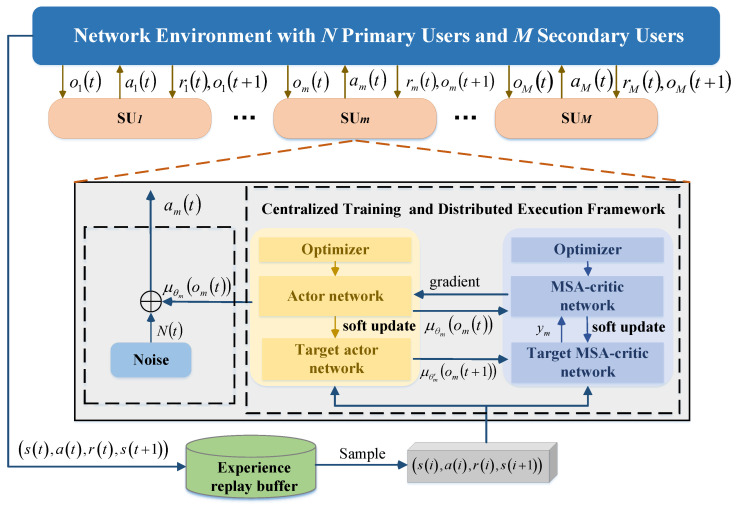
Schematic of multi-user opportunistic spectrum access based on centralized training with decentralized execution framework.

During the training phase, each agent is enabled to acquire global information, ensuring that the overall system performance is thoroughly taken into account during the decision-making process. In the execution phase, each agent relies only on local information for decentralized decision-making, which improves the scalability and real-time performance of the system. This method is particularly suitable for solving the complex interaction problem of SUs in channel selection and power control, which effectively captures and coordinates the interactions among users through shared global information, thus realizing multi-user decision-making in the same time slot. As shown in Figure 2, the system adopts a training mechanism based on experience replay, where each SU interacts with the environment. First, it obtains the local observation information of the current time slot and inputs it into the deep neural network model to generate the corresponding channel selection and power control decisions. After executing the decision, the SU receives feedback from the environment. During the interaction in each time slot, the generated experience samples are stored in the experience replay buffer. Network parameters are updated by randomly sampling mini-batches of experiences from the experience replay buffer in the training phase. Through this continuous process of interactive learning and policy optimization, SUs can gradually adjust their decision-making strategies, ultimately achieving global optimization of system performance.

### 4.1. Overview of DRL

In reinforcement learning (RL), the agent observes the current state $s\left(t\right)$ of the environment, takes an action $a\left(t\right)$ according to policy π, receives a corresponding reward $r\left(t\right)$, and transitions to the next state $s\left(t+1\right)$.

The classical RL algorithm Q-learning defines an action-value function, called the Q-function, with which the expected cumulative discounted reward of the agent is denoted as $Q^{\pi }\left(s,a\right)=E_{\pi }\left[\sum_{t=0}^{\infty }\eta^{t}r\left(t\right)\left|s\left(t\right)=s,a\left(t\right)=a\right.\right]$. The variable $\eta \in \left[0,1\right)$ represents the discount factor, which measures the importance of future rewards. The goal of RL is to find the optimal policy $\pi^{*}$ that maximizes the expected cumulative discounted reward. This can be described by the Bellman optimality equation as follows:

(9) $$Q^{*}\left(s,a\right)=E\left[r\left(t\right)+\eta \max_{a^{\prime }}Q^{*}\left(s\left(t+1\right),a^{\prime }\right)\left|s\left(t\right)=s,a\left(t\right)=a\right.\right],$$

where $\max_{a^{\prime }}Q^{*}\left(s\left(t+1\right),a^{\prime }\right)$ represents the maximum expected cumulative discounted reward obtained after taking the optimal action $a^{\prime }$ in the next state $s\left(t+1\right)$. The agent updates the Q-value using the following formula through its interaction with the environment:

(10) $$Q\left(s\left(t\right),a\left(t\right)\right)\leftarrow Q\left(s\left(t\right),a\left(t\right)\right)+\lambda \left[r\left(t\right)+\eta \max_{a^{\prime }}Q\left(s\left(t+1\right),a^{\prime }\right)-Q\left(s\left(t\right),a\left(t\right)\right)\right],$$

where $\lambda \in \left[0,1\right)$ is the learning rate.

The Q-learning algorithm performs well in scenarios with small state and action spaces. However, when faced with high-dimensional state or action spaces, its performance becomes constrained, making it challenging to effectively address more complex problems. To overcome this limitation, DRL combines the strengths of deep learning and RL by utilizing deep neural networks to approximate both the policy and the Q-function, thus enabling the effective handling of large-scale and complex problems. DQN utilizes deep neural networks to approximate the Q-value function, overcoming the limitations of classic Q-learning in high-dimensional spaces. DDPG separately approximates both the policy and the Q-value function, enabling it to more effectively handle problems with continuous action spaces.

### 4.2. State, Action and Reward for MADRL-OSA

In this paper, each SU is regarded as an agent. Each agent makes decisions based on its local observations and interacts with the environment through action selection. The joint actions of multiple agents collectively determine the state transition process and the acquisition of immediate rewards of the system. Agents maximize the global cumulative reward through strategic coordination and collaboration. Specifically, the process involves the state, action and reward function are defined as follows.

**State:** In a multi-agent environment, each agent can obtain an observation in each time slot, denoted as $o_{m}\left(t\right),m=1,2,\cdots ,M$. Specifically, the observation of each SU at time slot *t* includes the following information: the channel state $Z_{m}\left(t\right)=\left[z_{m1}\left(t\right),z_{m2}\left(t\right),\cdots ,z_{mN}\left(t\right)\right]$ sensed at time slot *t*, which is used to evaluate the availability of each channel, the gain $h_{S_{m}T,S_{m}R}\left(t\right)$ from its own transmitter to receiver, which reflects the fading characteristics of the signal transmission, the position $POS_{Tm}\left(t\right)=\left(pos_{Tmx}\left(t\right),pos_{Tmy}\left(t\right)\right)$ at the transmitter and the position $POS_{Rm}\left(t\right)=\left(pos_{Rmx}\left(t\right),pos_{Rmy}\left(t\right)\right)$ at the receiver, which are utilized to assess the spatial distribution and potential interference among users. Then, the observation of $SU_{m}$ at time slot is defined as

(11) $$o_{m}\left(t\right)=\left\{Z_{m}\left(t\right),h_{S_{m}T,S_{m}R}\left(t\right),POS_{Tm}\left(t\right),POS_{Rm}\left(t\right)\right\}.$$

The state of the multi-agent system includes the observations of all the agents, represented as

(12) $$s\left(t\right)=\left\{o_{1}\left(t\right),\cdots ,o_{m}\left(t\right),\cdots ,o_{M}\left(t\right)\right\}.$$

**Action:** The purpose of opportunistic spectrum access is to determine the channel selection and transmit power, then the action $a_{m}\left(t\right)$ of the $SU_{m}$ is defined as

(13) $$a_{m}\left(t\right)=\left\{c_{m}\left(t\right),p_{m}^{S}\left(t\right)\right\},m=1,2,\cdots ,M,$$

where $c_{m}\left(t\right)$ is channel selection and $p_{m}^{S}\left(t\right)$ denotes transmit power. Therefore, the set of actions of all SUs is represented as

(14) $$a\left(t\right)=\left\{a_{1}\left(t\right),\cdots ,a_{m}\left(t\right),\cdots ,a_{M}\left(t\right)\right\}.$$

The design of this action considers the dynamics and complexities of real-world communication scenarios. By optimizing both channel selection and power control, the agent can maximize spectrum utilization and enhance its communication performance while avoiding interference with PUs.

**Reward:** The design of the reward function is crucial in the opportunistic spectrum access problem. It not only directly influences the learning efficiency and policy optimization direction of the agent but also requires a comprehensive consideration of various factors, including spectrum utilization, SU communication performance, and overall system performance. In this paper, two reward functions are designed and compared in the simulation section. First, a sparse reward function is designed. When any SU collides with a PU, the reward is a negative penalty term ν. Conversely, when none of the SUs collide with the PU, the reward is the sum of the throughput of the SUs, denoted as $\sum_{m=1}^{M}R_{m}^{S}\left(t\right)$. This design focuses on factors such as collisions and throughput. It is simple and intuitive, effectively preventing interference from SUs to PUs. The sparse reward function expression is as follows:

(15) $$r_{m}^{\prime }\left(t\right)=\left\{\begin{array}{cc} \sum_{m=1}^{M}R_{m}^{S}\left(t\right), & NoCollision\\ -v, & Collision \end{array}\right.$$

Moreover, a multi-constraint dynamic proportional reward function is designed, which dynamically adjusts the reward proportion to accurately reflect the various constraints that SUs must satisfy during the access process. The reward function considers the interference between the SUs and the PUs and incorporates multidimensional factors such as channel competition and SNR constraints. This ensures that the agents receive more comprehensive and timely feedback signals during the training process, thereby guiding them to select more rational actions. The specific definition of the reward function is as follows:

(16) $$r_{m}^{\prime }\left(t\right)=\left\{\begin{array}{cc} \chi R_{m}^{S}\left(t\right), & \mathrm{if}\;pu\_collision=0\;\mathrm{and}\;n_{m,share}=1\;\mathrm{and}\;\gamma_{m}^{S}\left(t\right)⩾\gamma_{th}\\ \frac{R_{m}^{S}\left(t\right)}{n_{m,share}}, & \mathrm{if}\;pu\_collision=0\;\mathrm{and}\;n_{m,share}>1\;\mathrm{and}\;\gamma_{m}^{S}\left(t\right)⩾\gamma_{th}\\ -\nu , & \mathrm{if}\;pu\_collision=1\\ 0, & \mathrm{otherwise} \end{array}\right.$$

(17) $$r_{m}\left(t\right)=\sum_{m=1}^{M}r_{m}^{\prime }\left(t\right),$$

(18) $$r\left(t\right)=\{r_{1}\left(t\right),r_{2}\left(t\right),\cdots ,r_{m}\left(t\right)\}$$

where $r_{m}\left(t\right)$ and $R_{m}^{S}\left(t\right)$ are the reward and throughput of $SU_{m}$ in time slot *t*, respectively. pu_collosion is a flag indicating the collision status of the PU channel, where 1 means a collision between the SU and the PU, and 0 means no collision. When no collision occurs, $n_{m,share}$ represents the number of SUs sharing the channel, $\gamma_{m}^{S}\left(t\right)$ denotes the SNR of $SU_{m}$, $\gamma_{th}$ is the minimum SNR threshold, χ is the reward coefficient, and ν is the penalty term for collision. This reward function is designed from a global perspective, coordinating the actions of multiple SUs to maximize the overall throughput of the SUs. To achieve this, a shared reward mechanism is adopted, where all SUs share the same global reward value, $r\left(t\right)$, which is the sum of the individual reward values of all SUs. The multi-constraint dynamic proportional reward function is described in detail as follows:

(1) When an SU selects a channel that collides with the PU, a penalty term ν is applied, resulting in a negative reward. This design aims to inhibit interference from the SU to the PU, thereby protecting the communication quality of the PU.
(2) When multiple SUs select the same channel without colliding with the PU, and the SNR of all SUs meets the minimum requirement, the reward is $\frac{R_{m}^{S}\left(t\right)}{n_{m,share}}$. In this design, the more collisions between SUs, the lower the reward, which encourages the SUs to select the channel with less competition.
(3) When an SU does not collide with the PU or other SUs, and the SNR meets the required threshold, the reward is $\chi R_{m}^{S}\left(t\right)$. At this time, the SU is given higher rewards, and χ can be used to adjust the magnitude of the reward.
(4) In other instances, such as when the SNR of SU fails to meet the minimum requirement or when the SU does not select a channel, the reward is 0.

### 4.3. Multi-Agent Centralized Training with Decentralized Execution Framework

**Figure 3:**
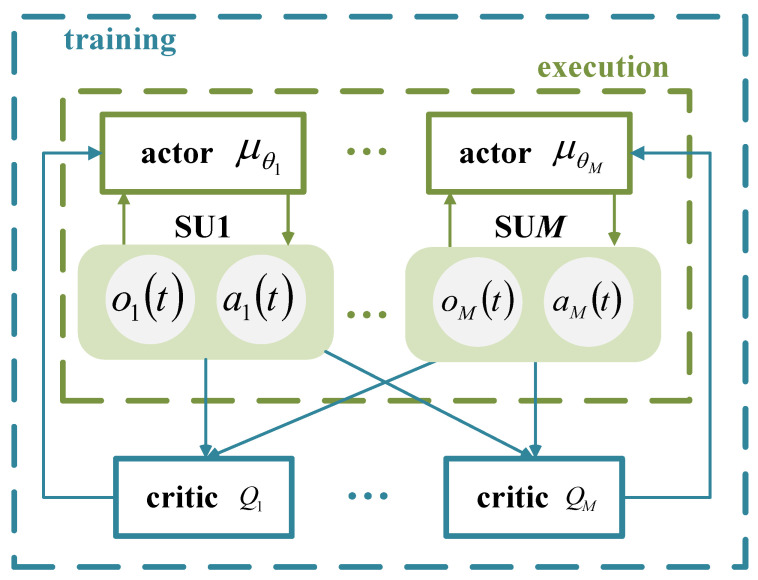
Schematic of the centralized training with decentralized execution framework for multiple SUs.

Each SU operates within a network based on the Actor–Critic (AC) framework, where the actor network generates actions based on the observations of the SU. The critic network provides feedback by evaluating the value of the actions, assisting the actor network in optimizing its policy. The centralized training with decentralized execution framework for multiple SUs is shown in Figure 3. During training, the input to the actor network is the observation of each SU, denoted as $o_{m}\left(t\right),m=1,2,\cdots ,M$, and the output is represented as $\mu_{\theta_{m}}\left(o_{m}\left(t\right)\right),m=1,2,\cdots ,M$. To improve the exploration capabilities of the agent, noise $N\left(t\right)$ is added to the output of the actor network, and the action of $SU_{m}$ is represented as $a_{m}\left(t\right)=\mu_{\theta_{m}}\left(o_{m}\left(t\right)\right)+N\left(t\right)$, where $N\left(t\right)$ decays with an increase in the training episodes. The input to the critic network consists of the observations and actions of all SUs, represented as $\left(s\left(t\right),a\left(t\right)\right)$, and the output is the action-value function $Q_{m}\left(s\left(t\right),a\left(t\right)\right)$. During execution, the input to the actor network is the observation $o_{m}\left(t\right),m=1,2,\cdots ,M$ of each SU, and the output is the action $a_{m}\left(t\right)=\mu_{\theta_{m}}\left(o_{m}\left(t\right)\right),m=1,2,\cdots ,M$ of the SU, with no exploration noise applied at this phase.

Each SU interacts with the environment as an agent. As shown in Figure 2, when $SU_{m}$ takes an action $a_{m}\left(t\right)$ based on observation $o_{m}\left(t\right)$, receiving a reward $r_{m}\left(t\right)$ and the next observation $o_{m}\left(t+1\right)$, these values are integrated into the experience entry $\left(o_{m}\left(t\right),a_{m}\left(t\right),r_{m}\left(t\right),o_{m}\left(t+1\right)\right)$ for that SU.

Subsequently, the experience entries of all SUs are merged into a multidimensional vector $\left(s\left(t\right),a\left(t\right),r\left(t\right),s\left(t+1\right)\right)$ and stored in the experience replay buffer to update network parameters. To enhance the stability of training and reduce the overestimation of action values by the critic network, the target network is introduced, including the target actor network and target critic network. The structure of the target network is the same as that of the corresponding actor network and the critic network.

The input of the target actor network is denoted as $o_{m}\left(t+1\right),m=1,2,\cdots ,M$, and the output is $\mu_{\theta_{m}}^{\prime }\left(o_{m}\left(t+1\right)\right),m=1,2,\cdots ,M$. The input of the target critic network is $\left(s\left(t+1\right),a^{\prime }\left(t+1\right)\right)$, and the output is $Q_{m}^{\prime }\left(s\left(t+1\right),a^{\prime }\left(t+1\right)\right)$.

When the network is updated, samples of mini-batch size are randomly selected from the experience replay buffer for training, denoting any set of samples as $(o_{m}\left(i\right),a_{m}\left(i\right),r_{m}\left(i\right)$, $o_{m}\left(i+1\right))$.

The desired output of the critic network is

(19) $$y_{m}=r_{m}+\eta Q_{m}^{\prime }\left(s\left(i+1\right),a^{\prime }\left(i+1\right)\right),$$

where η is the discount factor. The loss function is defined as

(20) $$L\left(\theta_{m}^{Q}\right)=\frac{1}{I}\sum_{i=1}^{I}{\left[r_{m}+\eta {Q^{\prime }}_{m}\left(s\left(i+1\right),a^{\prime }\left(i+1\right),\theta_{m}^{Q^{\prime }}\right)-Q_{m}\left(s\left(i\right),a\left(i\right),\theta_{m}^{Q}\right)\right]}^{2},$$

where $\theta_{m}^{Q}$ is the parameter of the critic network, and the optimizer is employed to update the parameter by minimizing the loss function according to the gradient descent method, as outlined below:

(21) $$\theta_{m}^{Q}\leftarrow \theta_{m}^{Q}-\lambda_{1}\nabla_{\theta_{m}^{Q}}L\left(\theta_{m}^{Q}\right),$$

where $\nabla_{\theta_{m}^{Q}}L\left(\theta_{m}^{Q}\right)$ denotes the gradient of the loss function with respect to $\theta_{m}^{Q}$ and $\lambda_{1}$ is the learning rate of the critic network.

The policy gradient of the actor network is represented as

(22) $$\nabla_{\theta_{m}}J\left(\mu_{\theta_{m}}\right)\approx \frac{1}{I}\sum_{i=1}^{I}\nabla_{\theta_{m}}\mu_{\theta_{m}}\left(o_{m}\left(i\right)\right)\nabla_{\mu_{\theta_{m}}\left(o_{m}\left(i\right)\right)}Q_{m}\left(s\left(i\right),a\left(i\right),\theta_{m}\right),$$

where $\theta_{m}$ is the parameter of the actor network which is updated using the policy gradient:

(23) $$\theta_{m}\leftarrow \theta_{m}+\lambda_{2}\nabla_{\theta_{m}}J\left(\mu_{\theta_{m}}\right)$$

where $\lambda_{2}$ is the learning rate of the actor network.

The parameters $\theta_{m}^{\prime }$ and $\theta_{m}^{Q^{\prime }}$ corresponding to the target actor network and target critic network, respectively, are updated using the soft update method as follows:

(24) $$\theta_{m}^{\prime }\leftarrow \alpha \theta_{m}+\left(1-\alpha \right)\theta_{m}^{\prime },$$

(25) $$\theta_{m}^{Q^{\prime }}\leftarrow \alpha \theta_{m}^{Q}+\left(1-\alpha \right)\theta_{m}^{Q^{\prime }},$$

where $\alpha \in \left(0,\left.1\right]\right.$ is the soft update coefficient.

## 5. MSA-MADRL-OSA Method

In the system model presented in this paper, the decisions of SUs are interdependent. The optimal action selection for each SU depends not only on its observations and actions but also on the significant influence of other behaviors of SUs. For example, when one SU selects a particular channel or adjusts its transmit power, it may interfere with the available channels and communication quality for other SUs, thus impacting the overall system performance. MADRL-OSA method, through centralized training with decentralized execution, can address the interdependence issue between agents to some extent. However, its critic network typically employs a simple multilayer perceptron (MLP) to process the observations and actions of all agents, which may struggle to effectively capture the complex dependencies between agents, especially when the number of agents is large or the environment is highly dynamic. Furthermore, the MLP cannot adaptively focus on information that is more important to the decision-making of the current agent, potentially leading to redundant or inefficient learning processes.

To address these limitations, we introduce a multi-head self-attention mechanism in the critic network of the MADRL-OSA method and propose the MSA-MADRL-OSA method. The MSA mechanism computes the similarities between SUs and dynamically assigns attention weights, allowing the network to capture the dynamic behaviors and intricate interactions of other SUs more effectively. This enables the SU to identify which other SUs have a greater impact on its current channel selection and transmit power, thereby improving the ability of the network to estimate action values.

### 5.1. Multi-Head Self-Attention

The attention mechanism is a computational model miming the human visual or cognitive process of selectively focusing on important information. It dynamically assigns weights to highlight key parts of the input data, thus enhancing the ability of the model to handle complex data. Common types of attention mechanisms include self-attention, channel attention, and spatial attention, each tailored for different tasks. Among them, MSA is particularly effective in capturing internal dependencies within sequences. By performing parallel computations with multiple attention heads, MSA extracts information from different subspaces, allowing it to more comprehensively capture the multidimensional interactions among multiple agents [28].

**Figure 4:**
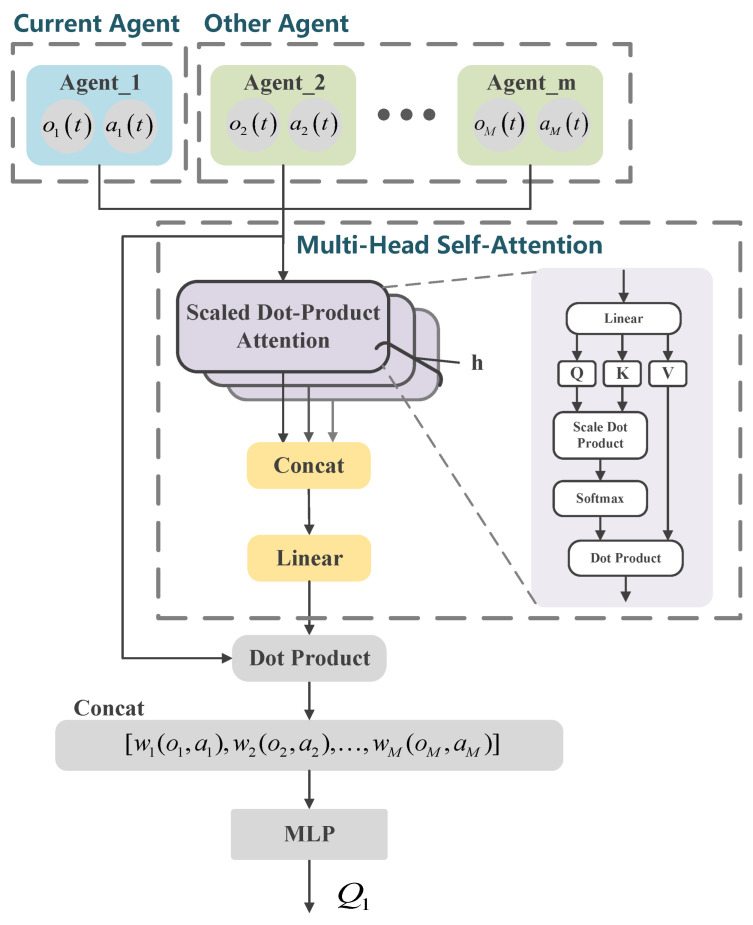
MSA network structure.

The core concept of the self-attention mechanism is to dynamically assign attention weights by calculating the relationships between the query, key, and value. Specifically, the query represents the target that requires attention, the key represents the features of other elements, and the value represents the actual information of those elements. Attention weights are typically computed using scaled dot-product attention, as illustrated in Figure 4. Given an input sequence $X\in R^{n\times d}$, the query matrix $Q=XW_{Q}$, key matrix $K=XW_{K}$, and value matrix $V=XW_{V}$ are generated through linear transformations, where $W_{Q}$, $W_{K}$, and $W_{V}$ are earnable weight matrices. The dot product of the query and key matrices, $QK^{T}$, is then computed and normalized by a scaling factor of $\sqrt{d_{k}}$ (where $d_{k}$ is the dimension of the key vector), yielding the attention scores. Finally, the attention scores are passed through a softmax function to produce the attention weight matrix.

(26) $$A\left(Q,K,V\right)=softmax\left(\frac{QK^{T}}{\sqrt{d_{k}}}\right).$$

This matrix represents the importance of each position in the sequence to the other positions. Finally, the attention weights are multiplied with the value matrix *V* to obtain the output O=AV of the self-attention mechanism, which incorporates the contextual information of all the positions in the sequence and serves as an input for subsequent processing.

The multi-head attention mechanism enhances the expressive power of the model by performing parallel calculations with multiple attention heads. Specifically, the Q, K, and V of the input sequence are each projected into low-dimensional spaces via *h* sets of distinct learnable linear projections, resulting in representations of dimensions $d_{Q}$, $d_{K}$, and $d_{V}$. After projection, each set of Q, K, and V undergoes independent attention function calculations, generating outputs of dimension $d_{V}$. These outputs are then concatenated and mapped back into the original $d_{\mathrm{model}}$ dimensional space using a learnable linear weight matrix $W^{O}$, yielding the final multi-head attention output. The computation formula for multi-head attention is as follows:

(27) $$MultiHead\left(Q,K,V\right)=Concat\left(head_{1},\ldots ,head_{h}\right)W^{O},$$

where the calculation for each attention head is

(28) $$MultiHead\left(Q,K,V\right)=Concat\left(head_{1},\ldots ,head_{h}\right)W^{O},$$

Here, $W_{i}^{Q}\in R^{d_{model}\times d_{k}}$, $W_{i}^{K}\in R^{d_{model}\times d_{k}}$ and $W_{i}^{V}\in R^{d_{model}\times d_{v}}$ are the projection matrices for the i-th attention head, and $W^{O}\in R^{hd_{v}\times d_{model}}$ is the output projection matrix.

### 5.2. Multi-Head Self-Attention for Critic

In the MADRL-OSA network described in Section 4, each agent has an independent critic network. The critic network evaluates the action value of the actions of the current agent in a given state, providing gradient information to optimize the policy of the actor network. To more accurately model the complex interactions among multiple agents, the critic network introduces an MSA mechanism to explicitly capture these interactions, enabling a more precise understanding of how other agents influence the decision-making of the current agent. As shown in Figure 4, the MSA–critic network takes the observations $o=[o_{1},o_{2},\ldots ,o_{M}]$ and actions $a=[a_{1},a_{2},\ldots ,a_{M}]$ of all agents as input and passes them through the MSA layer. The self-attention mechanism computes attention weights $\left[w_{1},w_{2},\ldots ,w_{M}\right]$ between the current agent and other agents, quantifying the degree of importance of each other agent on the current agent. These weights are then used to perform a weighted aggregation of the observations and actions of other agents, which are concatenated to form the context information for the current agent.

(29) $$c_{concat}=Concat\left(w_{1}\left(o_{1},a_{1}\right),w_{2}\left(o_{2},a_{2}\right),\ldots ,w_{M}\left(o_{M},a_{M}\right)\right),$$

Next, the context information $c_{concat}$ is fed into a multilayer perceptron (MLP), where it undergoes several non-linear transformations to yield the action-value estimate $Q_{m}$ for the current agent. The parameter update rule for the critic network follows the MADRL-OSA algorithm outlined in Section 4, as shown in Equations (21) and (22).

Based on the description of the MSA-MADRL-OSA method above, Algorithm 1 summarizes its implementation process.

**Algorithm 1** MSA-MADRL-OSA algorithm.
1**Initialize** neural networks for all agents and experience replay buffer.2**for** episode = 1 **to** maximum episodes **do**3   Initialize state $s\left(t\right)$.4   **for** time-slot t=1,2,⋯,T **do**5      Each $SU_{m}$ selects an action $a_{m}\left(t\right)=\mu_{\theta_{m}}\left(o_{m}\left(t\right)\right)+N\left(t\right),m=1,2,\cdots ,M$.6      Execute action, receive reward $r\left(t\right)$, and observe next state $s\left(t+1\right)$.7      Store $\left(o_{m}\left(t\right),a_{m}\left(t\right),r_{m}\left(t\right),o_{m}\left(t+1\right)\right)$ for each $SU_{m}$ in experience replay buffer.8      Update state: $s\left(t\right)=s\left(t+1\right)$.9      Sample a random mini-batch of *I* samples from experience replay buffer.10      **for** $SU_{m}$ = 1 **to** *M* **do**11         Update MSA-critic by minimizing the loss:         $L\left(\theta_{m}^{Q}\right)=\frac{1}{I}\sum_{i=1}^{I}{\left[r_{m}+\eta {Q^{\prime }}_{m}\left(s\left(i+1\right),a^{\prime }\left(i+1\right),\theta_{m}^{Q^{\prime }}\right)-Q_{m}\left(s\left(i\right),a\left(i\right),\theta_{m}^{Q}\right)\right]}^{2}$,         $\theta_{m}^{Q}\leftarrow \theta_{m}^{Q}-\lambda_{1}\nabla_{\theta_{m}^{Q}}L\left(\theta_{m}^{Q}\right)$.12         Update actor networks using the policy gradient:         $\nabla_{\theta_{m}}J\left(\mu_{\theta_{m}}\right)\approx \frac{1}{I}\sum_{i=1}^{I}\nabla_{\theta_{m}}\mu_{\theta_{m}}\left(o_{m}\left(i\right)\right)\nabla_{\mu_{\theta_{m}}\left(o_{m}\left(i\right)\right)}Q_{m}\left(s\left(i\right),a\left(i\right),\theta_{m}\right)$,         $\theta_{m}\leftarrow \theta_{m}+\lambda_{2}\nabla_{\theta_{m}}J\left(\mu_{\theta_{m}}\right)$.13         Update target network parameters:         $\theta_{m}^{\prime }\leftarrow \alpha \theta_{m}+\left(1-\alpha \right)\theta_{m}^{\prime }$,         $\theta_{m}^{Q^{\prime }}\leftarrow \alpha \theta_{m}^{Q}+\left(1-\alpha \right)\theta_{m}^{Q^{\prime }}$.14      **end for**15   **end for**16
**end for**



### 5.3. Computational Complexity

The computational complexity of the proposed MSA-MADRL-OSA algorithm primarily resides in the actor and critic networks, especially during the training phase. The actor network uses an MLP architecture, while the critic network combines MSA and MLP components. For the actor network, the computational complexity for a single forward pass is denoted as $O(d_{state}\cdot n_{1}+\sum_{l=1}^{L-1}n_{l}\cdot n_{l+1})$, where $d_{state}$ represents the input dimension (i.e., state dimension), and $n_{l}$ refers to the number of neurons in the l-th layer. The computational complexity of the MSA in the MSA–critic network is primarily made up of the following components: First, the input state and action information (with dimensions $d_{state}+d_{action}$) is linearly projected to generate the query, key, and value, which incurs a complexity of $O(3\cdot d_{agent}\cdot {\left(d_{state}+d_{action}\right)}^{2})$, where $d_{agent}$ represents the number of agents and $d_{action}$ is the action dimension. Next, the complexity for computing the attention scores and weighted sum is $O(2\cdot d_{agent}^{2}\cdot \left(d_{state}+d_{action}\right))$, as it requires calculating the dot product of the query and key for each agent, and then applying the attention weights to the value. Finally, the concatenated outputs from the multi-head attention are mapped back to the original space through a linear projection, which has a complexity of $O\left(d_{agent}\cdot {\left(d_{state}+d_{action}\right)}^{2}\right)$. Therefore, the total complexity for the multi-head self-attention mechanism is $O(4d_{agent}{\left(d_{state}+d_{action}\right)}^{2}+2d_{agent}^{2}\left(d_{state}+d_{action}\right))$. The MLP portion has a complexity of $O(\sum_{l=1}^{L-1}n_{l}\cdot n_{l+1})$ for a single forward pass. As a result, the overall computational complexity for one pass through the MSA–critic network is $O(4d_{agent}{\left(d_{state}+d_{action}\right)}^{2}+2d_{agent}^{2}\left(d_{state}+d_{action}\right)+\sum_{l=1}^{L-1}n_{l}\cdot n_{l+1})$.

At the execution phase, the MSA-MADRL-OSA algorithm relies solely on the actor network to generate actions. The forward propagation complexity for each inference is denoted as $O(d_{\mathrm{state}}\cdot n_{1}+\sum_{l=1}^{L-1}n_{l}\cdot n_{l+1})$, and its comparatively low overhead ensures efficient real-time decision-making. The proposed MSA-MADRL-OSA framework effectively balances computational complexity between the training and execution phases. By leveraging the parallel computing capabilities of GPUs, matrix operations are accelerated, significantly improving overall computational efficiency, thus enabling the framework to meet the stringent real-time requirements of multi-agent environments.

## 6. Results and Discussion

### 6.1. Simulation Setup

In this paper, the PyTorch framework (version 1.11.0, developed by Meta (formerly Facebook), and it was obtained from the official website https://pytorch.org/) is utilized to build a simulation test environment that implements opportunistic dynamic spectrum access for multiple PUs and SUs. In the simulation, the PUs and SUs are randomly distributed within a 160 m × 160 m area, with the transmitter and receiver distance between each user ranging from 25 to 45 m. The PUs are fixed in position, while the SUs move randomly within the designated area in each time slot. The arrival rate of the PU in the channel is set to 0.4 by default. The number of PUs is set to 6 by default, and the number of SUs is set to 3. The transmit power of the PUs varies randomly between 40 mW and 100 mW, while the transmit power of the SUs is selected between 5 mW and 20 mW. Other simulation parameters are shown in Table 1. To better align with practical applications, this paper further considers perturbations in realistic environments and their impact on system performance. To this end, several experiments are designed to demonstrate the advantages of the proposed method under these conditions. First, we consider the simulation of the competition among secondary users due to spectrum constraints in limited spectrum resources by varying the number of primary and secondary users. Second, we simulate the variation of throughput performance under different PU arrival rates, aiming to demonstrate the dynamic adjustment capability of the system. Finally, we also simulate a sudden change in the primary user arrival rate to model a realistic scenario of resource congestion and drastic changes in channel occupancy. Through these simulation setups, we comprehensively evaluate the spectrum access strategy in complex, dynamic environments, particularly under perturbations and channel state change conditions, thereby verifying the adaptability and robustness of our method across different scenarios.

In the MSA-MADRL-OSA method proposed in this paper, the actor network consists of three fully connected layers, each containing 64 neurons. The MSA–critic network comprises one MSA layer and three fully connected layers. By default, the MSA layer has 8 heads, with the hidden layers containing 128 neurons. All fully connected layers also have 128 neurons, and each fully connected layer is followed by an activation function tanh to produce the output. Each agent independently builds its actor network and MSA–critic network, along with the corresponding target networks, all sharing the same architecture. During training, a mini-batch size is randomly sampled from the replay buffer for each iteration. The training consists of multiple episodes, each fixed to 100 time steps. At each time step, the immediate reward is calculated based on the reward function, and the cumulative reward is computed at the end of the episode. The proposed method is tested on an i7-7700K CPU (Intel, located in Santa Clara, CA, USA) and an RTX-3060Ti GPU (Intel, located in Santa Clara, CA, USA). The average inference time of each agent is approximately 0.59 ms, meeting real-time communication requirements. All experimental results are based on the average of 10 independent trials to ensure the reliability of the data.

To evaluate the performance of the MSA-MADRL-OSA method, we compared it with four different methods. The first two methods are ablation experiments of the MSA-MADRL-OSA method, aimed at analyzing the impact of the proposed MSA–critic and the multi-constraint dynamic proportional reward function on the training results.

- MADRL-OSA: The MADRL method described in Section 4, where the only difference from MSA-MADRL-OSA is that the critic network uses the standard MLP layers without an MSA layer. All other network architectures and simulation settings are identical to those of MSA-MADRL-OSA.
- MSA-MADRL-OSA SR: This method employs the sparse reward (SR) function described in Section 4.2, with all other network architectures and simulation settings the same as those of MSA-MADRL-OSA.
- MADQN: Multi-agent deep Q-learning network. The distributed DQN is used for action selection, and all other simulation settings are identical to those of MSA-MADRL-OSA.
- RANDOM: Random action selection method, where actions are chosen randomly. All other simulation settings are identical to those of MSA-MADRL-OSA.

### 6.2. Method Performance Analysis

**Figure 5:**
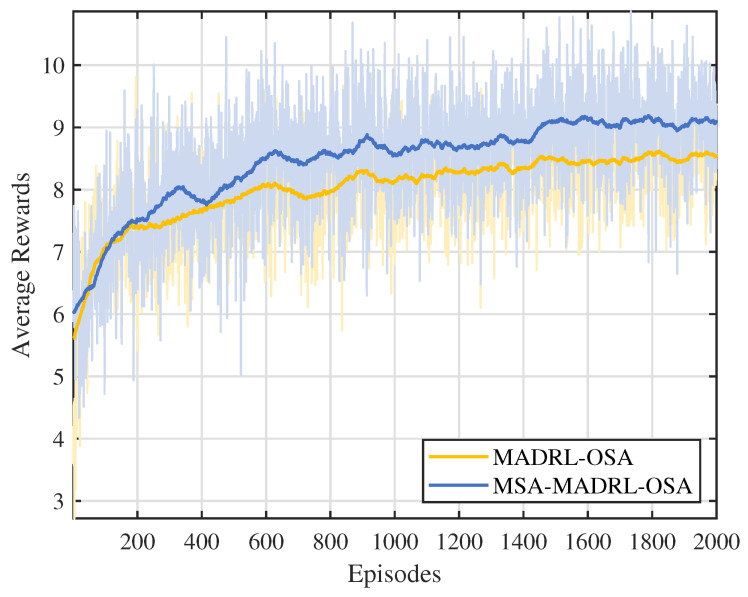
Average rewards under different methods.

Figure 5 shows the trend of average cumulative rewards over episodes during the training process for the MSA-MADRL-OSA method and the comparison method. To more clearly display the variation pattern, the curves in the figure are smoothed using a moving average (MA) with a window size of 100, while the shaded area reflects the fluctuation range of the original data before smoothing. As can be seen from Figure 5, the proposed MSA-MADRL-OSA method achieves convergence within 2000 episodes, and the average cumulative reward after convergence significantly outperforms the comparison method. This performance improvement is mainly attributed to the MSA mechanism introduced in the critic network, which enhances the ability of the network to estimate action values, thereby improving the rationality of action selection and further optimizing the learning efficiency of the policy.

**Figure 6:**
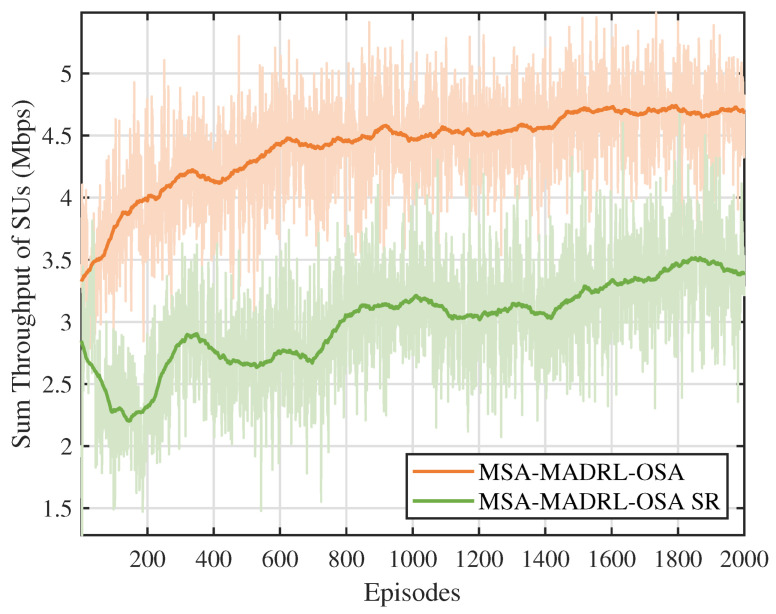
Sum throughput of SUs under different reward functions.

Figure 6 compares the throughput performance of the multi-constraint dynamic proportional reward function proposed in this paper with the sparse reward function. To ensure a fair comparison, both reward functions are evaluated using the MSA-MADRL-OSA method. As shown in Figure 6, the proposed reward function outperforms the sparse reward function in terms of both training stability and throughput after convergence. This is because the sparse reward function primarily focuses on channel collisions and overall throughput, neglecting other crucial constraints that SUs must satisfy during access, such as SNR requirements and channel contention. This oversight often leads the agent to fall into local optima when optimizing the policy. In contrast, the proposed reward function dynamically adjusts the reward coefficients based on changes in the environmental state, overcoming the limitations of a static reward mechanism. Thus, it effectively enhances the decision-making performance of the agent.

In addition, we also explored the hyperparameters of the MSA–critic network. By parallel computing multiple attention heads, MSA captures feature information from different subspaces, thereby enhancing the ability of the agent to understand the states and behaviors of other agents in the environment. As a result, the number of attention heads, denoted as *h*, is a crucial hyperparameter, and its value has a significant impact on the performance of the algorithm. As outlined in the simulation setup, the experimental results presented in this paper were obtained with the default value of h=8. To further investigate the effect of *h*, we conducted the same experiment with *h* values of 2, 4, 8, and 16. Table 2 presents the average rewards and throughput after convergence from 10 independent experiments. The results show that as *h* increases, the obtained rewards gradually improve, particularly when h=8, where both the reward and throughput reach their highest levels. Overall, under all settings of *h*, MSA-MADRL-OSA consistently outperforms MADRL-OSA in terms of reward. Therefore, it can be concluded that the proposed MSA-MADRL-OSA method demonstrates strong robustness across a wide range of *h* values, achieving superior performance.

### 6.3. Adaptability to Complex Environments

**Figure 7:**
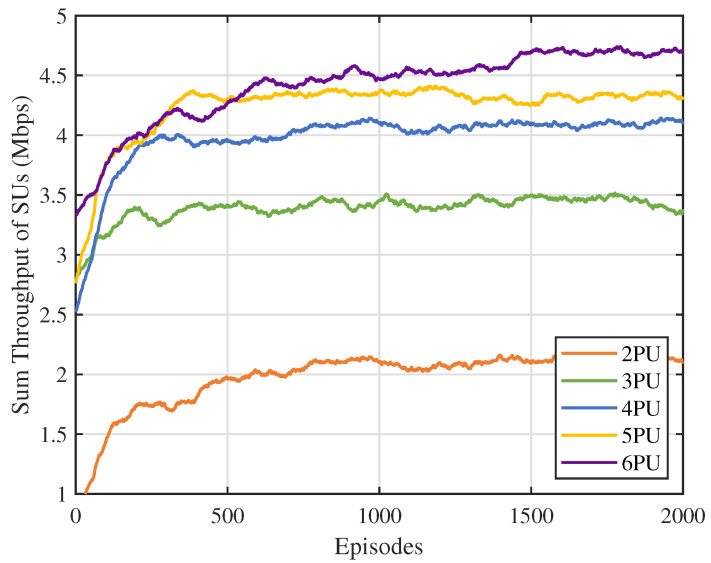
Number of PUs vs. sum throughput of SUs.

**Figure 8:**
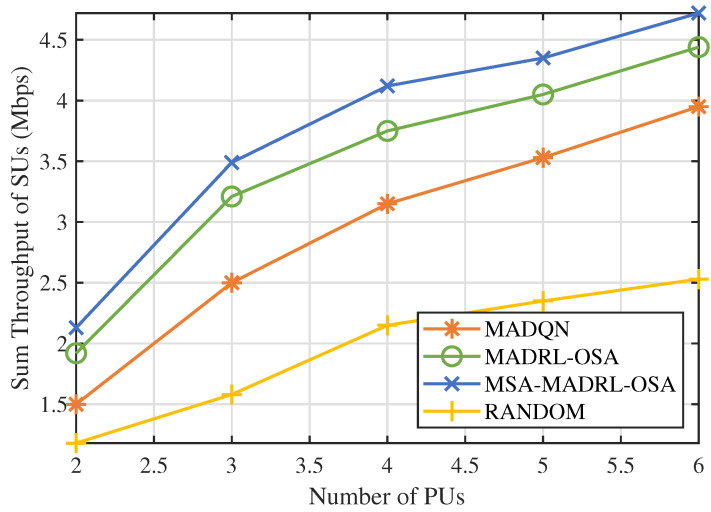
Number of PUs vs. sum throughput of SUs by different methods.

First, we analyzed the impact of the number of channels N on the performance of the MSA-MADRL-OSA method. Figure 7 illustrates the variation in the sum throughput of SUs under different values of N. The experimental results show that the method converges stably within 2000 episodes for any value of N, indicating that its convergence ability is not affected by the increase in the number of channels, thus highlighting its excellent scalability. Figure 8 compares the sum throughput of SUs with the number of PUs under different methods. As the number of channels increases, the sum throughput of SUs improves for all methods. However, when N becomes large, the rate of throughput increase gradually slows down. This occurs because, as channel resources increase, SUs can distribute more evenly across different channels, causing more SUs to approach their SNR limits, which constrains throughput growth. Nevertheless, the experimental results demonstrate that the MSA-MADRL-OSA method significantly outperforms other comparison methods across all channel configurations. Compared to the second-best-performing method, MSA-MADRL-OSA achieves an average improvement of 8.29% in sum throughput for SUs. The MSA-MADRL-OSA method dynamically adapts to changes in the number of channels and by optimizing channel selection and power control policies, it maintains high throughput performance in complex environments, demonstrating exceptional scalability and adaptability.

**Figure 9:**
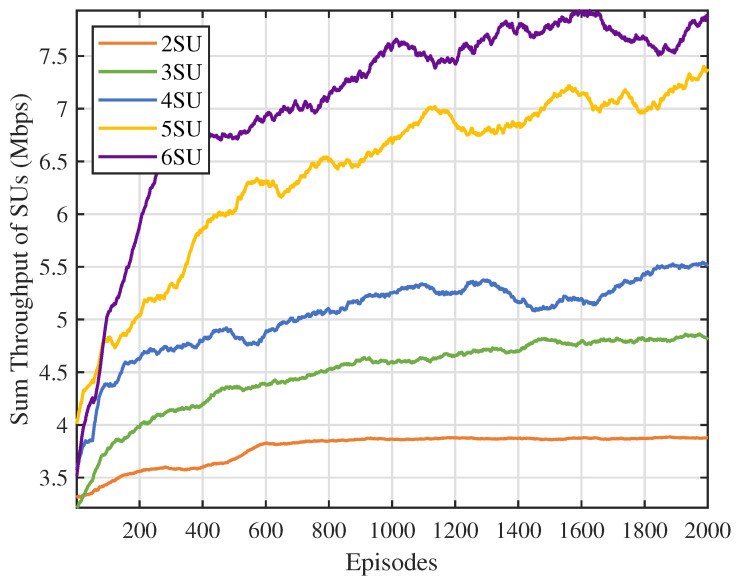
Number of SUs vs. sum throughput of SUs.

**Figure 10:**
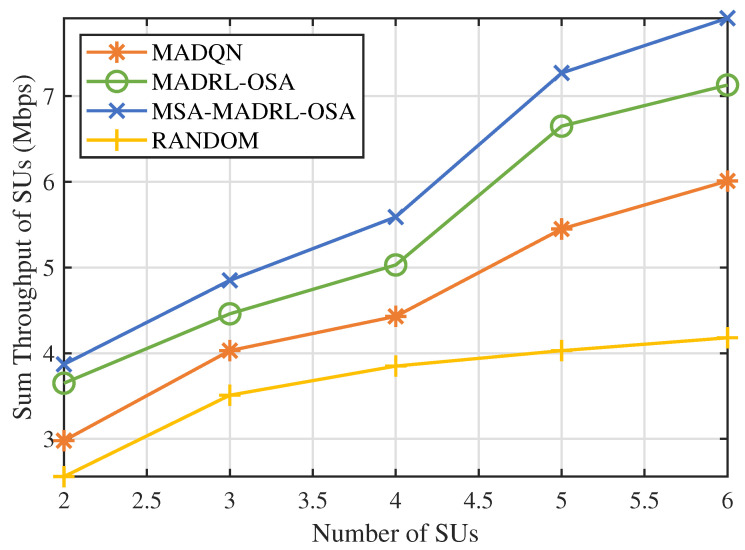
Number of SUs vs. sum throughput of SUs by different methods.

Next, we varied the number of SUs to evaluate the performance of the MSA-MADRL-OSA method under different network densities. Figure 9 and Figure 10 show the sum throughput of SUs using the MSA-MADRL-OSA method, with a PU arrival rate of 0.2, for different numbers of SUs, as well as the relationship between sum throughput and the number of SUs for various methods. As shown in Figure 9, the MSA-MADRL-OSA method remains able to converge effectively as the number of SUs increases. In Figure 10, as more SUs join the network, the sum throughput for all methods increases. However, as network density grows, interference between SUs also increases, which impacts their normal transmission and leads to transmission failures in some time slots. As a result, the rate of increase in sum throughput gradually slows down. It is worth noting that both algorithms that centralized training with decentralized execution framework outperform the MADQN algorithm with the distributed architecture, consistently achieving higher summed SU throughput with varying numbers of SUs. With the help of MSA, the MSA-MADRL-OSA algorithm achieves an average improvement of 9.54% in sum throughput for SUs compared to the second-best-performing method. This result highlights the strong adaptability of the proposed method to collisions and resource contention in high-density user scenarios and its ability to maintain high performance under challenging conditions, demonstrating excellent robustness.

**Figure 11:**
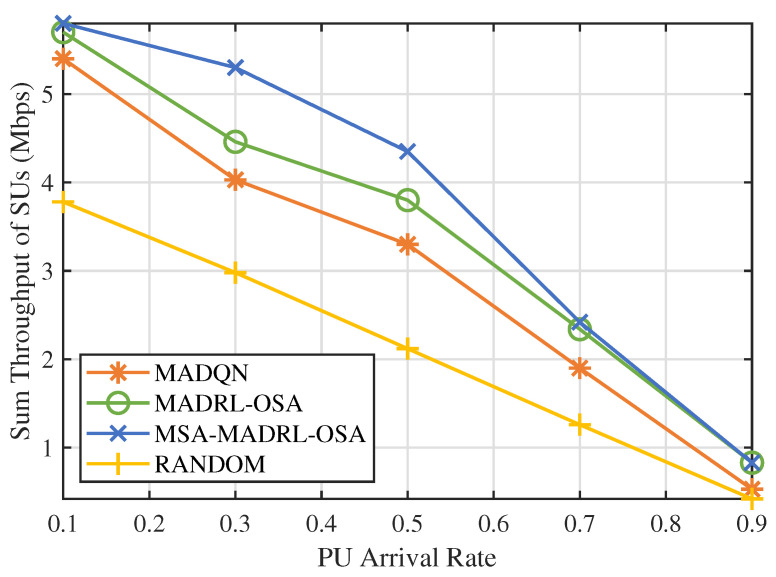
PU arrival rate vs. sum throughput of SUs by different methods.

In practical environments, the activity patterns of PUs are dynamic, with their arrival rates influenced by various environmental factors. Figure 11 shows the sum throughput comparison of four methods under different PU arrival rates. The RANDOM method exhibits a linear decrease in throughput as the arrival rate increases, due to the SUs randomly selecting channels and power. MADQN, MADRL-OSA, and MSA-MADRL-OSA exhibit similar performance at low and high arrival rates. This similarity arises primarily because, at high arrival rates, PUs occupy the channels for extended periods, thereby reducing access opportunities for SUs. Conversely, at low arrival rates, SUs encounter fewer challenges in making accurate decisions, resulting in improved performance across three methods. At moderate arrival rates, the differences between the three methods become more evident. MADQN performs poorly, while MADRL-OSA and MSA-MADRL-OSA are better at adapting to changes in arrival rates. In particular, MSA-MADRL-OSA, through the multi-head attention mechanism, enhances the flexibility and accuracy of the policy, resulting in the best throughput. At arrival rates of 0.3 and 0.5, the throughput of MSA-MADRL-OSA is 18.8% and 14.5% higher than that of MADRL-OSA, respectively. This experiment validates the advantages of MSA-MADRL-OSA, which is enhanced by the multi-head attention mechanism, in complex environments.

### 6.4. Robustness in Dynamic Scenarios

**Figure 12:**
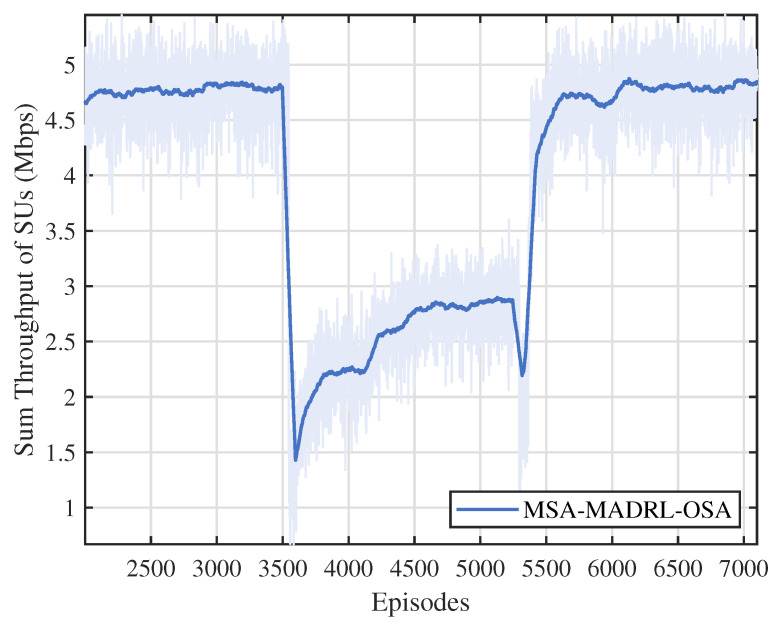
Throughput performance under changes in channels.

To further evaluate the robustness and adaptability of the proposed method, we implemented the MSA-MADRL-OSA approach in a dynamically changing environment. As shown in Figure 12, during the initial phase, the PU arrival rate is set to 0.4, and after training, the method converges with a throughput of approximately 4.7 Mbps. At episode 3500, the PU arrival rate abruptly increases to 0.6. This sudden change results in higher channel occupancy, leading to resource congestion and increased interference among SUs, which causes a noticeable decrease in throughput. During this period, the network dynamically adjusts channel selection and power control based on observations, optimizing channel and power settings. Following this adjustment, throughput gradually increases and stabilizes at around 2.9 Mbps. At episode 5300, when the PU arrival rate returns to 0.4, throughput experiences a brief dip but quickly recovers to its normal level. The experiment, which tests the proposed method under increasing and decreasing arrival rates, demonstrates that the method effectively addresses challenges arising from the sudden number of available channel changes, exhibiting excellent adaptability and robustness.

## 7. Conclusions

In this paper, an MSA-MADRL-OSA method is proposed to enhance the sum throughput of SUs in dynamic spectrum access environments. The proposed method optimizes spectrum access for multiple SUs based on centralized training with decentralized execution framework and designs a multi-constraint dynamic proportional reward function to guide the SUs to make more rational action choices. At the same time, to further optimize the spectrum access policy, an MSA mechanism is introduced into the deep neural network. Through computational complexity analysis, the proposed MSA-MADRL-OSA method in the execution phase relies solely on the actor network to generate actions. Its computational complexity is closely related to the state dimension. The comparatively low overhead ensures efficient real-time decision-making. The simulation results demonstrate that the proposed method significantly outperforms the comparison method in terms of average cumulative reward after convergence and exhibits superior performance across different numbers of channels and SUs. Compared with the second-best-performing method, the proposed method achieves an average enhancement of 8.29% in the sum throughput of SUs under varying numbers of channels and 9.54% under different numbers of SUs. Moreover, the MSA-MADRL-OSA method effectively addresses the challenges posed by sudden changes in the number of available channels, demonstrating remarkable adaptability and robustness. This paper provides a new solution to the multiple-SU opportunistic spectrum access problem. Future work can further explore the application of this method in more complex network environments and combine it with other advanced intelligent algorithms to develop a more efficient and flexible spectrum access policy. This will offer new perspectives and methods for optimizing opportunistic spectrum access policy in cognitive radio networks.

## Figures and Tables

**Table 1:** Simulation parameter settings.

Parameter Name	Average Reward
Bandwidth *W*	1 MHz
Noise Power Spectrum Density $N_{0}$	$1\times 10^{-14}$ mW/Hz
Constant Term of Path Loss $C_{0}$	0
Path Loss Exponent β	22.7
Frequency Decay Factor $B_{\xi }$	20
Path Loss at Reference Distance $\xi_{0}$	41
Rician Factor *k*	8
The SINR Threshold for SU $\gamma_{th}^{S}$	−10 dB
**Hyperparameter**	**Value**
Learning Rate for Actor Network $\lambda_{1}$	$1\times 10^{-4}$
Learning Rate for Critic Network $\lambda_{2}$	$1\times 10^{-3}$
Discounted Factor η	0.95
The Experience Replay Buffer Capacity *D*	10,000
Time Step Length for Each Episode *T*	100
Mini-batch Size	256
Optimizer	Adam

**Table 2:** Rewards and throughput with different numbers of attention heads.

Different Methods	Average Reward	Sum of Throughput (Mbps)
2 Head	8.83	4.58
4 Head	8.91	4.62
8 Head	**9.12**	**4.72**
16 Head	8.67	4.54
MADRL-OSA	8.57	4.46

## Data Availability

Data are contained within the article.
